# Poly (Glycerol Sebacate)-Based Bio-Artificial Multiporous Matrix for Bone Regeneration

**DOI:** 10.3389/fchem.2020.603577

**Published:** 2020-11-23

**Authors:** Bo Liang, Qiang Shi, Jia Xu, Yi-Min Chai, Jian-Guang Xu

**Affiliations:** Department of Orthopedic Surgery, Shanghai Jiao Tong University Affiliated Sixth People's Hospital, Shanghai, China

**Keywords:** poly (glycerol sebacate), bio-artificial multiporous matrix, regeneration, vascularization, VEGF

## Abstract

In recent years, bone repair biomaterials that combine cells and bioactive factors are superior to autologous and allogeneic bone implants. However, neither natural nor synthetic biomaterials can possess all desired qualities such as strength, porosity, and biological activity. In this study, we used poly (glycerol sebacate) (PGS), a synthetic material with great osteogenic potential that has attracted more attention in the field of tissue (such as bone tissue) regeneration owing to its good biocompatibility and high elasticity. It also has the advantage of being regulated by material synthesis to match the bone tissue's strength and can be easily modified to become functional. However, pure PGS lacks functional groups and hydrophilicity. Therefore, we used PGS as the substrate to graft the adhesive ligands RGD and vascular endothelial growth factor mimetic peptide. The bone repair scaffold can be prepared through photo crosslinking, as it not only improves hydrophobicity but also promotes vascularization and accelerates osteogenesis. Simultaneously, we improved the preparation method of hydrogels after freeze-drying and crosslinking to form a sponge-like structure and to easily regenerate blood vessels. In summary, a bone repair scaffold was prepared to meet the structural and biological requirements. It proved to serve as a potential bone-mimicking scaffold by enhancing tissue regenerative processes such as cell infiltration and vascularization and subsequent replacement by the native bone tissue.

## Introduction

Bone repair following bone damage caused by trauma and disease is a complex process necessitating the need to identify better ways to promote bone regeneration (Shen et al., 2016). Various synthetic biomaterials, such as poly (lactic-co-glycolic acid) and polymethyl methacrylate, were designed to fill bone defects and promote bone regeneration, resulting in its repair. These biomaterials are popular because they circumvent the limited bone resources and avoid the immunological rejections associated with autogenic and allogeneic bone transplantations. They are also easy to design and more economic (Miri et al., 2016). Many studies combine bioactive factors or cells to design these synthetic biomaterials to compensate for the lack of bioactivity in synthetic biomaterials. These studies consider the osteogenic properties of the materials, such as excellent mechanical properties, biodegradability, good biocompatibility, and the osteogenic differentiation ability of stem cells. Several studies consider the relevance of vascularization and osteogenesis as the survival of transplanted cells and tissues in bone regeneration requires a microenvironment with a vibrant vascular network for nutrition delivery and waste removal. The cells in the center of the scaffold may undergo apoptosis since nutrition and oxygen around the tissue are often limited to a depth of 150–200 μm around the porous scaffold (Suresh and West, 2020). Therefore, novel biomaterials with angiogenic and biodegradable properties to support tissue integration and angiogenesis, with elastic properties to enhance bone tissue repair in a dynamic environment, are required. For better vascularization of biomolecules [e.g., vascular endothelial growth factor (VEGF) (Schnettler et al., 2003) and basic fibroblast growth factor (Stähli et al., 2015)], some metal ions (e.g., copper, cobalt, strontium, magnesium, boron, and calcium) have been used to promote angiogenesis. However, the use of VEGF is the most effective. The VEGF polypeptide not only recapitulates the function of the VEGF factor but also has advantages such as being economic and easily combining with materials through covalent binding to biomaterials (Hung et al., 2019).

VEGF is a potent cytokine secreted by bone cells. It can specifically bind to the VEGF receptor on the surface of endothelial cells to activate the signaling cascade, which eventually leads to the formation of new capillaries, a process called angiogenesis.

Therefore, distributions of VEGF ligands in biomaterials are crucial determinants of angiogenesis. Significant effort is required when combining the VEGF peptide with biomaterials to ensure satisfactory angiogenesis. Hydrogel microspheres covalently bonded peptide derived from vascular endothelial growth factor receptor 2 can mimic the ability of natural extracellular matrix through reversible binding of VEGF. The results showed that VEGF was released in a controlled manner, which promoted the growth and the tube formation of human umbilical vein endothelial cells (HUVECs) (Impellitteri et al., 2012). In another study, the authors designed a proteolytic degradable polyethylene glycol (PEG) hydrogel (García et al., 2016) loaded with VEGF. The hydrogel could achieve protease degradation dependent VEGF protein release while maintaining high VEGF bioactivity. In a critical-sized bone defect model, VEGF's delivery enhanced the infiltration of blood vessels into the defect. Other factors used in combination with VEGF can also promote blood vessels. Rufaihah et al. (2017) demonstrated that PEG-fibrinogen hydrogel provides good mechanical support and simultaneously releases two vascular-related factors, VEGF and angiopoietin 1, in myocardial remodeling to achieve recovery after myocardial infarction.

Poly (glycerol sebacate) (PGS) is a biodegradable synthetic polymer made of the two-block copolymer formed with the polycondensation of glycerol and sebacic acid. PGS elastomers have been successfully used in various biomedical fields because of their good biocompatibility, intentional elastic properties, and their abundant hydroxyl side groups that are easy to be functionalized (Kemppainen and Hollister, 2010; Luginina et al., 2020; Touré et al., 2020; Wang et al., 2020). Multiple osteogenic signals, such as growth factor injection and stem cell transplantation, have been introduced to PGS systems to achieve better bone recovery. Bioglass® 45S5 porous scaffolds produced via the replica foam technique (Chen et al., 2006) have also been coated with PGS to produce flexible and toughened scaffolds for bone tissue engineering (Chen Q. Z. et al., 2010). Synthesis and fabrication of porous, elastomeric nanocomposite scaffolds using biodegradable PGS and osteoinductive nanosilicates have been reported. The combination of elasticity and tailorable stiffness, tunable degradation profiles, and the osteoinductive capability of the scaffolds offer a promising approach for bone tissue engineering (Kerativitayanan et al., 2017). Lee et al. (2013) described a PGS elastomer containing stromal cell-derived factor 1 alpha, which can enhance the recruitment of endothelial cells and mesenchymal stem cells in the elastic vascular scaffolds by delivering stromal cell-derived factor 1 chemokine, proving that the composite stent has an ability to induce *in situ* vascular regeneration. Lee et al. (2009) reported a type of porous three-dimensional PGS scaffold with high elasticity. The scaffold had good biocompatibility and anticoagulant properties. This scaffold can be used to study the role of endothelial cells and other cells in the development of complex vascular tissue. However, at present, these studies focus on the osteogenic properties of PGS, and few of them combine the osteogenic and vascularization properties of PGS to study the final osteogenic effect. Therefore, it is worth exploring the effect of this combination of VEGF peptide and PGS biomaterials on osteogenesis.

To obtain good angiogenic ability, the material should have a porous sponge structure, which regulates cell ingrowth and makes the blood vessel easily integrated. Fabrication techniques can often determine the physicochemical and biological properties of the scaffold, including porosity, mechanical strength, osteo-conductivity, and bone regenerative potential. Many researchers have opted for electrospinning, salinization, and polymer dissolution methods to obtain porous material. However, these methods' limitations include extremely small pore sizes, complex preparation processes, or harmful dissolved residues. To overcome these problems, numerous investigators have proposed mechanisms for increasing the average pore size of scaffolds. Phipps et al. (2012) used three different technologies to adjust the pore size of PCL/col I/nanoHA hydrogel. Addition of the poly(ethylene oxide) polymer chain before the gel formation resulted in an increased pore diameter. Biological experiments show that this method can promote the regeneration of bone and vascular tissue by increasing cell invasion. Therefore, pore size and architecture play an important role in tissue regeneration. Meanwhile, a previous study has shown that a pore size range of 75–175 μm is believed to be appropriate for the ingrowth of chondrocytes and osteoblasts *in vivo* (Ansari et al., 2019). In light of these biophysical and biochemical cues, we were able to fabricate a PGS-based bio-artificial multiporous matrix via photopolymerization of an acrylate mainly comprising three building blocks: sebacic acid, glycerol, and oligo (acrylic acid) (Nguyen and West, 2002; Browning et al., 2015). Each of the major building blocks has proven to be biocompatible for bone regeneration (Sawhney et al., 1993; Kim et al., 2008). We hypothesized that by adjusting the pore size of PGS hydrogels containing VEGF mimetic peptide, some biological effects such as the speed and location of blood vessels and the quality of bone formation could also be affected, presenting a bone repair material that can rapidly induce the growth of new blood vessels *in vivo* and thereby improve bone repair.

## Materials and Methods

### Matrix Synthesis

Acr-PEG-QK peptide (a 15-mer VEGF mimetic peptide: Ac-KLTWQELYQLKYKGI-NH2) and Acr-PEG-RGD peptide were custom prepared by a commercial manufacturer (GLS, Shanghai, China) and supplied at 95% purity. Acr-PEG-QK peptide activity was verified by testing endothelial cell activity. PGS hydrogel was fabricated in two steps: (1) the PGS pre-polycondensation step was implemented as previously reported (Samourides et al., 2020), and (2) acrylate was chemically grafted on the PGS prepolymer according to previously published methods (Pashneh-Tala et al., 2020). For the poly condensation process, equimolar mixtures (1 M) of glycerol and sebacic acid were reacted at 120°C under argon for 24 h, followed by pressure reduction from 1 Torr to 40 mTorr for another 5 h. All chemicals were purchased from Sigma Aldrich unless stated otherwise. The PGS prepolymer was acrylated in the following manner: 20 g of PGS prepolymer and 200 mL of anhydrous dichloromethane were added into a dry round bottom flask. After the polymer completely dissolved, the mixture in the reaction bottle was cooled to 0°C under a positive pressure of nitrogen. Acryloyl chloride was slowly added to an equal molar amount of trimethylamine and stirred at 0°C for 24 h. The mixture was filtered, precipitated with ether, dialyzed, and lyophilized.

Hydrogels consisting of adhesive ligands RGD and VEGF mimetic peptide were generated through free radical polymerization of the double bond under ultraviolet light with photoinitiator Irgacure 2959. Non-functional matrices PGS was set as the control. In summary, Acr-PGS was dissolved in phosphate-buffered saline (PBS) at 10% (wt/vol) with 2.8 μM Acr-PEG-RGD, 80 μg/mL Acr-PEG-QK, and 0.05% Irgacure 2959 (Ciba) photoinitiator. Polymer solutions were crosslinked through exposure to a 365-nm ultraviolet light at 10 mW/cm^2^ for 10 min.

### Matrix Characterization

The hydrophilicity of PGS could be improved by introducing hydrophilic protein fragments into the polymer network. Therefore, the hydrophilicity of the crosslinked network was determined using the contact angle. The contact angle measuring instrument (Phoenix 300) was used to measure the contact angle of water in two groups, with and without protein fragments (*n* = 3). The samples were then converted into a 0.5 mm-thick film. Pure water (4 μL) was deposited on the surface of the material. The drop image was captured, and the tangent line was measured after the contact angle was stable.

The hydrogel samples were freeze-dried, the initial weight (W0) was measured, and then they were immersed in PBS buffer at 37°C to measure the swelling ratio. After water swelling, the hydrogel samples were taken out after 24 h, with water on the surface removed. We also recorded the swelling mass. When the mass was balanced, the weight was set to Ws. The water uptake by these samples was determined with Equation (1):

(1) $$\begin{array}{r} \frac{Ws-W0}{Ws}\times 100 \end{array}$$

The evaluation of degradation performance was conducted using the PBS buffer at 37°C and a speed of 80 rpm for 90 days. At specific time intervals, the immersed samples were taken out of the buffer, and weighed (Wf) after overnight lyophilization. The mass loss of samples was calculated with Equation (2):

(2) $$\begin{array}{r} \frac{W0-Wf}{W0}\times 100 \end{array}$$

### *In vitro* Characterization of rBMSCs and HUVECs in Composite Scaffolds

This study was performed in strict accordance with the National Institutes of Health Guide for the Care and Use of Laboratory Animals. We isolated rat bone marrow stromal cells (rBMSCs) from the femur for *in vitro* experiments. HUVECs were purchased from Shanghai Institutes for Biological Sciences (Shanghai, China). We seeded rBMSCs on different scaffolds to observe the adhesion of cells to the material. Briefly, the 3–5 generations' stem cells were cultured in Minimal Essential Medium supplemented with 10% fetus bovine serum (Gibco) and 1% antibiotics (penicillin/streptomycin, Gibco, USA). The cells were cultured in a moist chamber with 5% CO_2_ at 37°C for ~3 days and then digested with trypsin (0.05% trypsin/EDTA, Gibco). Serum was added to the culture medium to stop the digestion, and the cells were re-suspended in the medium. We placed 200 μL of cell suspension comprising 5 × 10^5^ cells/mL on different scaffolds in 24-well plates. The cells were cultured on the material for 1 h to adhere, and then 800 μL of the medium was added to submerge the material. rBMSCs were cultured on the scaffold for 3 days to observe the cell morphology. After removing the culture medium, they were rinsed with PBS three times and then fixed with 4% paraformaldehyde for 15 min. After rinsing with PBS three times, the cells were infiltrated with 0.5% Triton X-100 for 10 min and rinsed again with PBS two times. The cells were stained with fluorescein isothiocyanate-labeled phalloidin (Thermo Fisher Scientific, MA, USA) at 25°C for 1 h to mark the cytoskeleton. Cell morphology was observed using three-dimensional reconstruction of confocal images.

The proliferation rate of rBMSCs and HUVECs cultured for 1, 3, and 7 days was obtained using the MTT assay (Invitrogen). TCPS surface was set as the positive control group. Briefly, different scaffolds were placed into a 24-well plate, and 3 × 10^5^/well rBMSCs were seeded on the surface of the scaffold and TCPS.

HUVEC migration on the scaffolds was observed using confocal microscopy 72 h after the initial cell seeding. After rinsing three times in PBS, the hydrogels were fixed in 4% paraformaldehyde at 25°C for 15 min, embedded in paraffin, and sectioned at 4.5 mm with a microtome. The slides from the middle of the hydrogels were stained with DAPI (Invitrogen) and observed under a laser scanning confocal microscope (Leica, Germany). At least three parallel experiments were conducted for each sample.

### Osteogenic Differentiation

To detect the effect of different materials on the osteogenic differentiation of stem cells, we seeded rBMSCs with a density of 3 × 10^4^ cells/well on different materials placed in 24-well plates for 3 and 7 days to detect the expression of osteogenic related genes. At that point, Trizol method was used to split the cells and extract RNA. The content of RNA was determined using spectrophotometry (Thermo Scientific, MA, USA). For reverse transcription, we used a reverse transcription kit (Takara) and customized primers (Sangon biotech, Shanghai) to perform quantitative polymerase chain reaction tests to analyze the osteogenic differentiation of rBMSCs further. Glyceraldehyde 3-phosphate dehydrogenase was used as the reference gene. The sequence of the primer pairs used was as follows: GAPDH: forward: 5′-CAGGGCTGCCTTCTCTTGTG-3'; reverse: 5'-AACTTGCCGTGGGTAGAGTC-3'; RUNX 2: forward: 5'-CCTTCCCTCCGAGACCCTAA-3'; reverse: 5'-ATGGCTGCTCCCTTCTGAAC-3'; ALP: forward: 5'-ACCGCAGGATGTGAACTACT-3'; reverse: 5'-GAAGCTGTGGGTTCACTGGT; OCN-3'; forward: 5'-ATTGTGACGAGCTAGCGGAC-3'; reverse: 5'-GCAACACATGCCCTAAACGG-3'. The results were expressed as 2^−ΔΔ*Ct*^. Alkaline phosphatase (ALP) staining was conducted after 7 days to observe the ALP activity of each group.

### Micro Perfusion, Micro-Computed Tomography Imaging, and Tissue Section Staining

All animal procedures were approved by the Animal Research Committee of the Sixth People's Hospital, Shanghai Jiao Tong University. Distal femoral defect models were established using Sprague–Dawley rats weighing 300–400 g. The surgical operations were conducted on rats anesthetized intraperitoneally with pentobarbital. A 1.0–1.5 cm sagittal incision on the side of the knee joint was made, and a 2.5-mm defect was drilled using a Kirschner wire (American fine science tool company, Fine Science Tool, SFO, USA). The scaffold (Ø2.5 × 3 mm) was then implanted. Nine rats with 18 defects were allocated to the following three groups: control (*n* = 6), PGS-P (*n* = 6), and L-PGS-P (*n* = 6). Two weeks after the operation, the rats in each group were administered 0.9% saline + 4 mg/mL papaverine (Sigma Aldrich, Darmstadt, Germany) after inhalation anesthesia, and then 0.9% saline and 10% neutral formalin. After fixation, 30 mL of 80% (vol/vol) diluted mv-122 microfilament (Flowtec) was injected into the aorta with a syringe and an indwelling needle. After polymerization overnight, the samples were removed. The data from the scans were evaluated with a micro-computer tomography reconstructed using the VG studio to establish three-dimensional models. The percentage of new bone volume relative to tissue volume and vascular volume/total implant volume in the bone defect was quantified using the auxiliary histomorphometric software and CTvox software. For microscopic examination of the newly formed bone and vessels, decalcified samples were stained immunohistochemically.

### Statistical Analysis

Data were analyzed using Origin 8.0 (OriginLab, MA, USA). All experiments were performed thrice (*n* = 3), and results are presented as mean ± standard deviation. A non-parametric test two-way analysis of variance with multiple comparisons was used for significance testing. The significance was calculated at 95% confidence interval with the two-tail alpha level of 0.05; p < 0.05 was considered significant.

## Results

### PGS-Based Bio-Artificial Multiporous Matrix Fabrication and Characteristics

**Figure 1:**
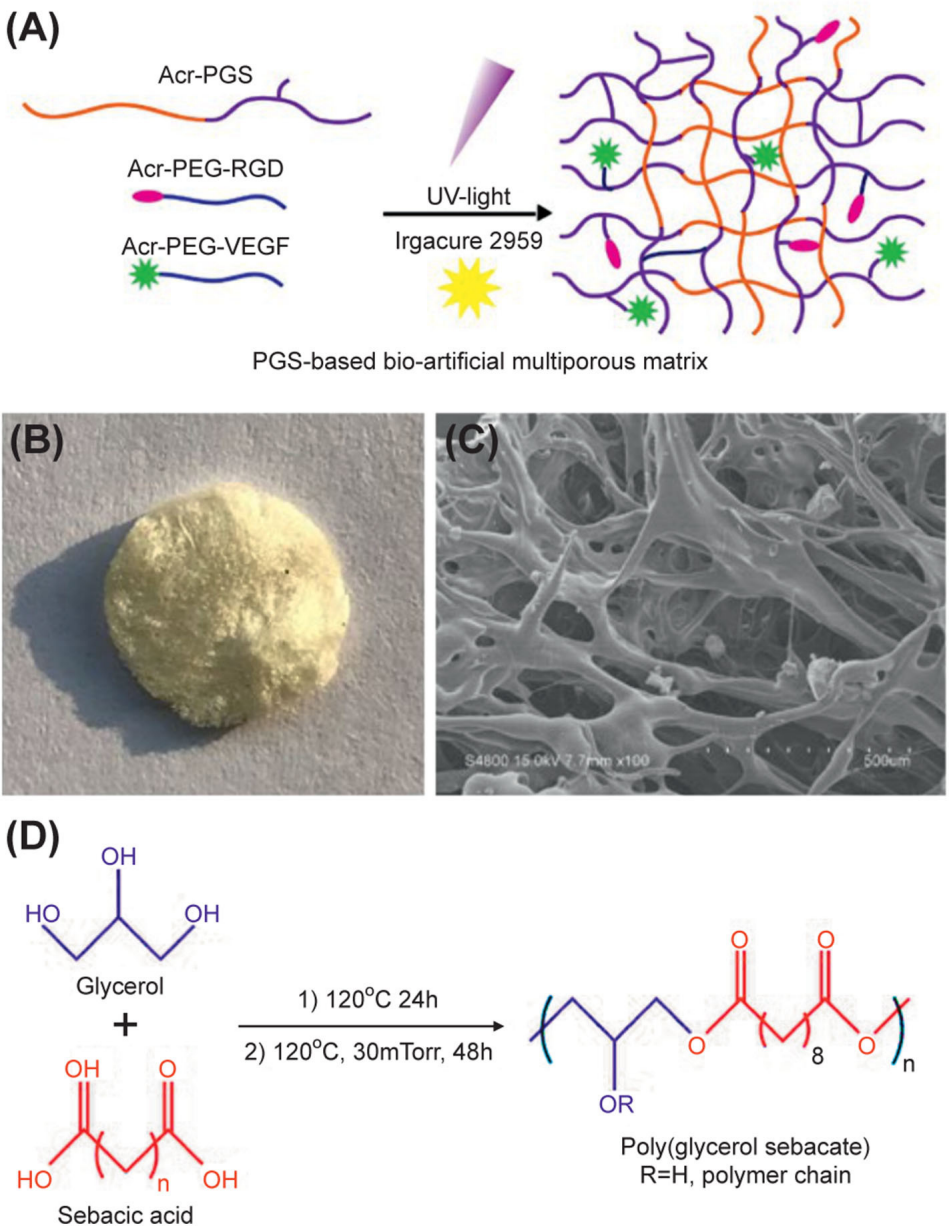
Design of PGS-based bio-artificial matrices. **(A)** Bioactive ligands RGD peptide, QK peptide are mono-PEG-acrylated for covalent crosslinking into PGS network. Addition of a photoinitiator to PGS aqueous solution of PGS followed by 10 min UV irradiation results in crosslinking of hydrogel through free radical polymerization of acrylate end groups before and after lyophilization. **(B)** Gross image of L-PGS-P. **(C)** SEM image of L-PGS-P. **(D)** Synthesis of poly (glycerol sebacate) polymer.

The first step in the process of *in situ* induced bone regeneration is cell migration and invasion. We engineered bio-artificial multiporous hydrogel matrices consisting of RGD adhesive ligands and the VEGF mimetic peptide for better cell invasion and vascularization (Figure 1A). Hydrogel matrices consisting of Acr-PEG-RGD and Acr-PEG-QK peptides were generated through free radical polymerization of acrylate end-functional groups with low-intensity ultraviolent light using photoinitiator Irgacure 2959 (Figure 1A) (named as PGS-P). We freeze-dried the PGS-P solution before crosslinking (named as L-PGS-P) for the formation of porous structures. We obtained the porous structure from L-PGS-P, as seen in the gross image and scanning electron microscopy image (Figures 1B,C) and the PGS pre-polycondensation step was implemented (Figure 1D).

**Figure 2:**
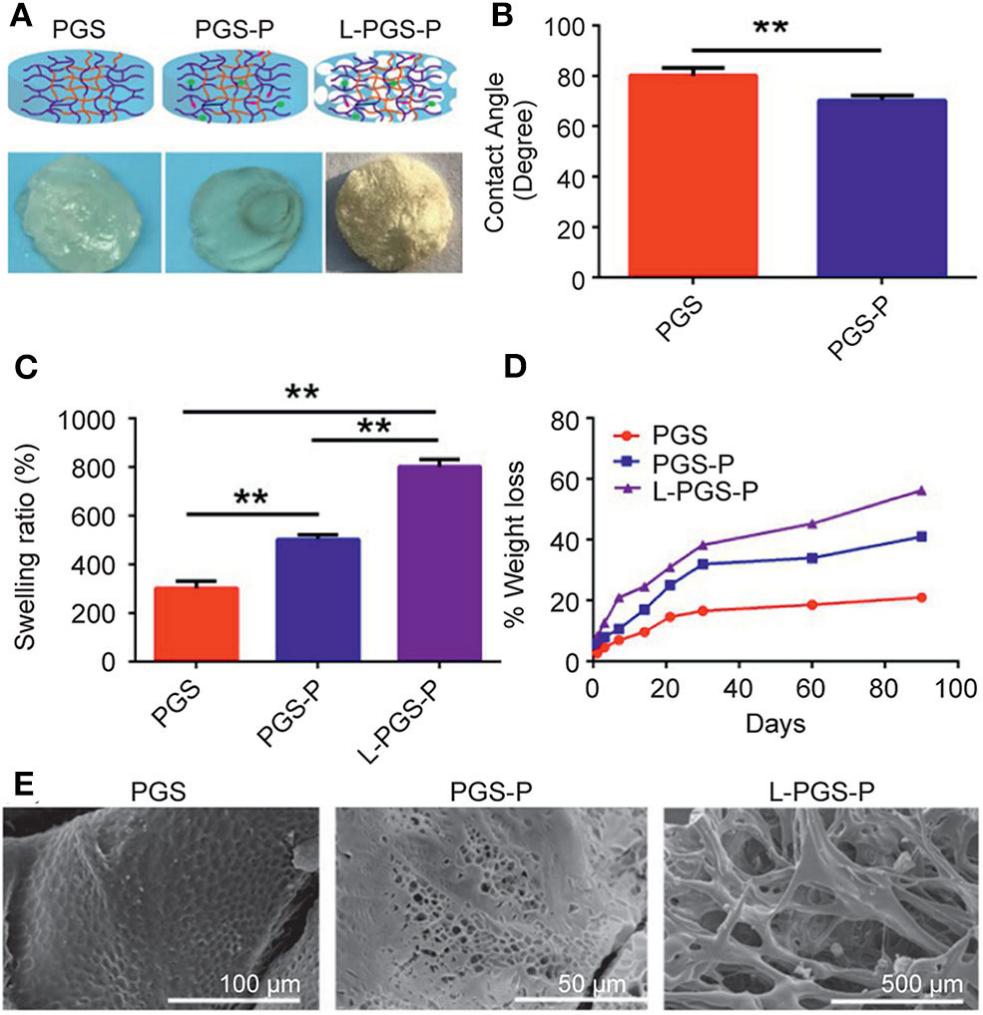
Physical properties of different scaffolds. **(A)** Macroscopic view of PGS, PGS-P, and L-PGS-P scaffolds. **(B)** Water contact angle was performed on PGS and PGS-P. **(C)** Swelling ratio (**p < 0.01 significance to P for 24 h). **(D)** Degradation performance of PGS, PGS-P, and L-PGS-P scaffolds. **(E)** SEM image of PGS, PGS-P, and L-PGS-P.

On the macroscopic view of the PGS, PGS-P, and L-PGA-P matrices, we found that the PGS matrix was opaque, while the PGS-P matrix was transparent. L-PGS-P presented as a porous sponge sample after freeze-dried pretreatment (Figure 2A). The hydrophilic property of biomaterials is a very important physical and chemical property. It can directly affect cell adhesion and biocompatibility, hence its application in biomedicine (Chen S. et al., 2010; Gaharwar et al., 2011, 2012). The addition of protein motifs within the PGS crosslinked network promoted hydrophilic characteristics to the PGS prepolymer. We investigated surface hydrophilic characteristics of the PGS and PGS-P matrices using a contact angle measuring instrument (Phoenix 300) (Figure 2B). Pure PGS hydrogels showed a contact angle of 80 ± 1.7 degrees with the water. As expected, the addition of protein motifs (PGS-P) increased the hydrophilic nature reducing the contact angle to 71 ± 0.1 degrees. The swelling study was used to further evaluate the bulk hydration characteristic of PGS and PGS-P (Figure 2C). The swelling test revealed the maximum swelling ratio in the L-PGS-P matrix, while PGS water uptake was the least (^**^p < 0.01). The degradation rate is very important for tissue regeneration because it needs to match the tissue regeneration rate. Degradation products also need to be non-toxic to tissues. Therefore, the degradation rate was studied to evaluate if the polymeric materials are suitable for biomedical applications (Sheng et al., 2019; Dintcheva et al., 2020). The *in vitro* degradation of the PGS, PGS-P, and L-PGS-P matrices was investigated using the PBS buffer at 37°C over a 90-day period (Figure 2D). The mass loss of all samples was linear and non-sudden disintegration related to erosion degradation of PGS matrix from the surface. The addition of protein motifs and multiporous architecture resulted in a faster degradation rate. Figure 2E shows that the porosity and pore size were improved in the L-PGS-P matrix, with pore sizes being 200–500 μm.

### *In vitro* Characterization of rBMSCs and HUVECs in Composite Scaffolds

**Figure 3:**
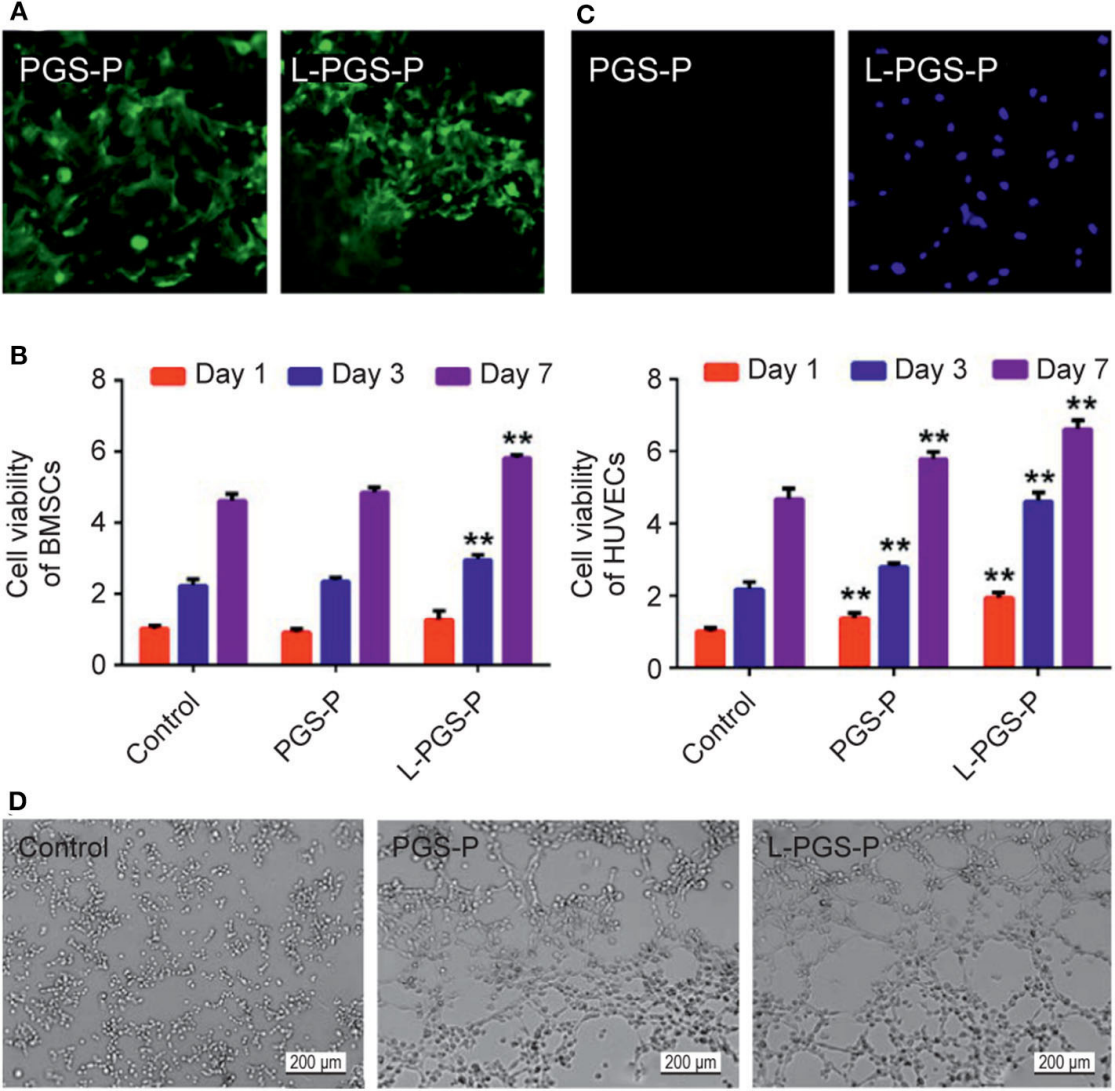
Cellular behavior on different scaffolds. **(A)** Cell adhesion activity of PGS-P and L-PGS-P scaffolds illustrated in a 40x magnification picture. **(B)** The proliferation rate of the two kinds of adhered cells on day 1, 3, and 7 was tested using an MTT assay. **(C)** Cell infiltration was observed by DAPI staining. L-PGS-P scaffold supports cell infiltration of HUVECs *in vitro*; 20x magnification picture was captured at cross section. **(D)** Representative images of tube formation for 6 h with different medium from scaffolds soak liquid (soak for 7 days). **P < 0.01, compare with control group at different time point.

There is little information about the improvement of L-PGS-P in the angiogenic microenvironment. Through the cytotoxic test, we found that both the PGS-P composite scaffold and L-PGS-P porous composite had good biocompatibility (Figure 3B). rBMSCs adhered well to their surfaces (Figure 3A). For cell infiltration and angiogenic activity, there was more HUVECs entry into L-PGS-P (Figure 3C) and significantly increased endothelial tube formation of HUVECs (Figure 3D) *in vitro*. We speculate that these results may be due to the multiporous architecture of L-PGS-P and more VEGF release after 7 days of soaking in the medium (Figure 2D).

### Osteogenic Differentiation

**Figure 4:**
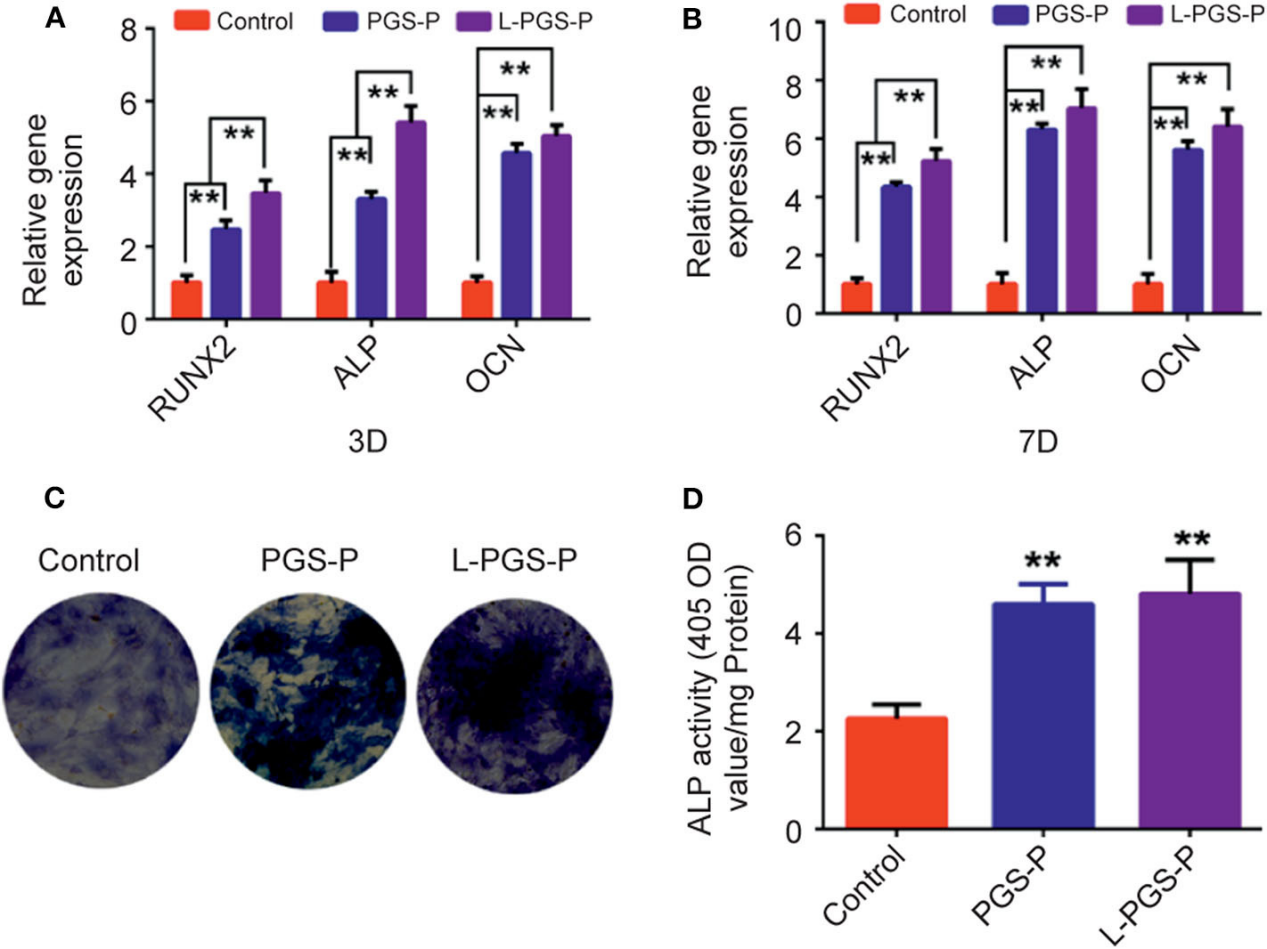
The osteogenic effects induced by PGS-P and L-PGS-P on rat bone mesenchymal stem cells. **(A,B)** The statistics of RT-qPCR results for RUNX2, ALP, and OCN gene expression levels which were normalized to GAPDH after 3 and 7 days of osteogenic treating. **(C)** rBMSCs cultured on different scaffolds in osteogenic medium for 7 days were stained for ALP examination. **(D)** ALP activity was assessed by quantitative method. **P < 0.01, compare with control group.

To investigate whether bio-artificial matrix works on the osteogenic potential of rBMSCs, an analysis of its osteogenic ability was performed using RT-qPCR. Compared with the PGS-P scaffold, the mRNA expression levels of Runx2, ALP, and OCN in the L-PGS-P group were significantly higher (Figure 4A). These results agree with the ALP activity test. The trend of ALP activity on day 7 *in vitro* analysis of BMSCs showed more ALP activity in the L-PGS-P group after ALP staining (Figures 4B,C).

### Micro Perfusion, Micro-Computed Tomography Imaging, and Tissue Section Staining

**Figure 5:**
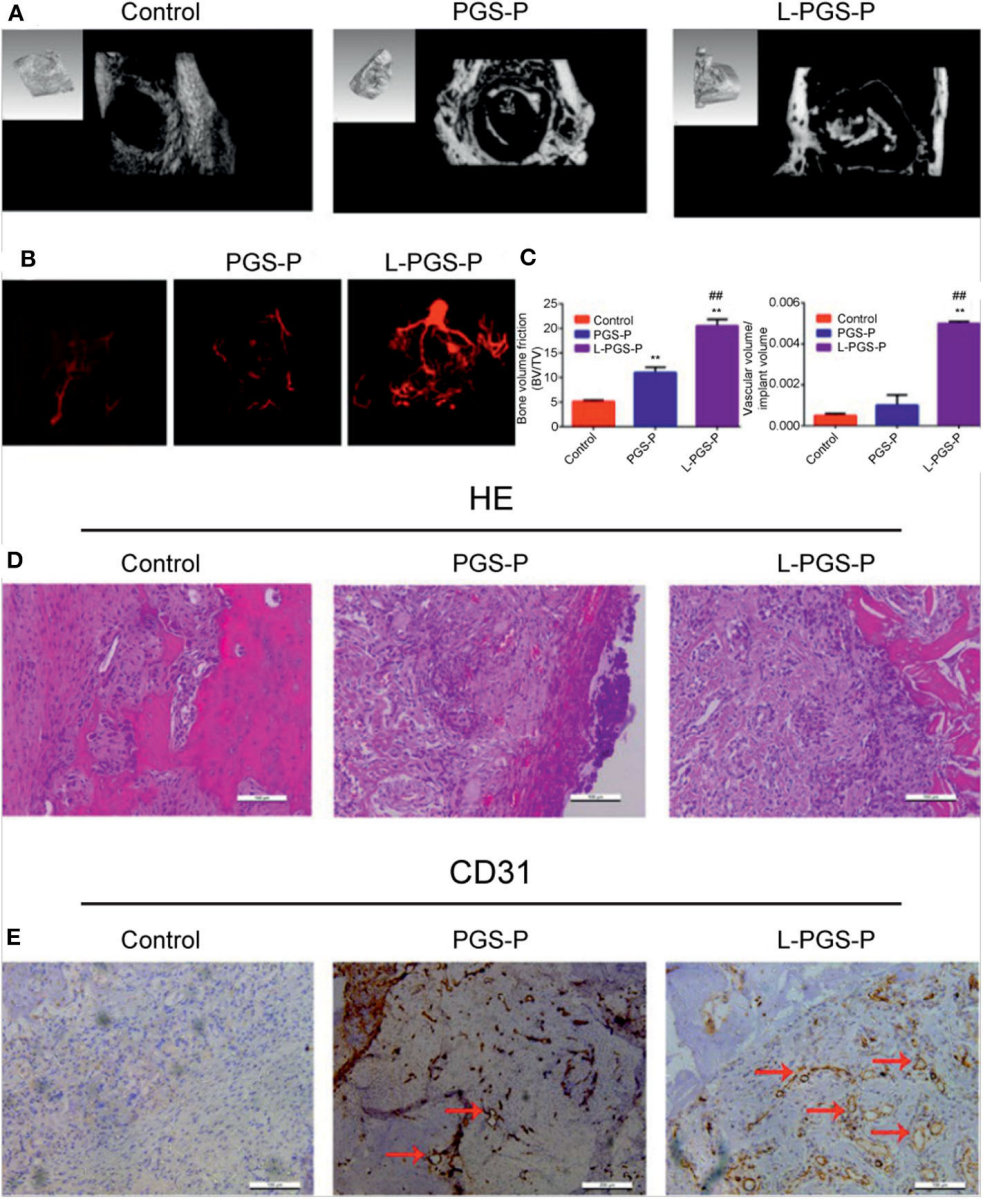
The proangiogenic and osteogenic performance of PGS-P and L-PGS-P scaffolds *in vivo*. **(A)** Representative computed tomography reconstruction images of the defects shows abundant mineralized tissues in L-PGS-P than PGS-P at 2 weeks after implantation. **(B)** Micro-computed tomography images of vessel in rat distal femur defects perfused with Microfil radio-opaque contrast agent. L-PGS-P implants showed vasculature in surrounding tissue growing into implant, but not in the PGS-P and control groups. **(C)** Bone volume fraction and quantification of vascular volume/total implant volume. **(D,E)** haematoxylin and eosin **(D)** and CD31 staining **(E)** was performed at 2 weeks after implantation to evaluate the vessel and bone formation. Remarkably, more mineralized tissue and CD31-positive cells were found in L-PGS-P than PGS-P. Red arrows indicate positively stained areas of CD31-positive blood vessels. **P < 0.01, compare with control group; ^*##*^*P* < 0.01, compare with PGS-P group.

To inspect the ability of L-PGS-P bio-artificial matrices to improve cell infiltration and vascularization, Microfil perfusion was used to evaluate vessel ingrowth in scaffolds (Figure 5B). By week 2, micro-computed tomography analysis of the scanned constructs showed animals receiving L-PGS-P exhibited an increase in perfusion to the defect compared with the PGS-P and control groups. Importantly, there were a few vessels within the periphery of the PGS-P scaffold. In contrast, larger and more regular vessels were growing into and around the L-PGS-P implants. We also quantified greater blood vessel density in defects treated with L-PGS-P than with PGS and empty controls (Figure 5C). The trends of osteogenic performance were paralleled with the results of vascularization (Figure 5A). These results agree with our *in vitro* test results, indicating better cell invasion in the L-PGS-P group (Figure 2C). Hematoxylin and eosin staining revealed pronounced fibrous tissue around the defect in the control group, and many blood vessels appeared around the defect and a few inflammatory cells around the material in the PGS-P group. In contrast, in the L-PGS-P group, a large proportion of the trabecular bone was produced in the defect (Figure 5D). The proangiogenic potential of the scaffolds was confirmed with CD31 immunostaining. Compared with limited staining in the control group, there were sufficient CD31-positive cells in the PGS-L and L-PGS-P groups. Interestingly, CD31 positive cells in the PGS-P group formed very narrow tubes but those in the L-PGS-P had very thick tubes (Figure 5E).

## Discussion

Vascularization of biomaterials is an essential step in bone healing and regeneration, as it can provide oxygen and nutrients for osteoblasts to survive and metabolize (Karageorgiou and Kaplan, 2005; Ekaputra et al., 2011). Therefore, tissue engineering pays increased attention to the vascularization ability of biomaterials in design. At the same time, in addition to providing biological clues for vascularization, scaffolds should be designed with pore sizes large enough to allow tissue infiltration and vascular growth. There are many manufacturing technologies to improve the pore size of hydrogels and promote tissue regeneration. However, the pores are not connected and the pore size is not large enough. Therefore, it is very important to adopt new methods to obtain larger pore size hydrogel scaffolds to facilitate vascular ingrowth and osteoblast infiltration in the whole material.

In this study, we chose PGS as the matrix because of its good mechanical properties and biological safety; further, its polymer structure is easy to be functionalized (Chen et al., 2011). Importantly, the ester bond of the PGS backbone can be hydrolyzed and degraded by enzymes *in vivo* (Wang et al., 2003); thus, with the degradation of the main chain, functional groups can be released and used (Phelps et al., 2010). However, the hydrophobicity of PGS is a limiting point and must be solved first (Crapo and Wang, 2010). We found that PGS can improve the hydrophobicity of PGS after functional peptide grafting without additional synthetic modification, which not only functionalizes the scaffold biological but also solves the problem that hydrophobicity makes it impossible to prepare hydrogels. Second, the hydrogel directly prepared is the same as most hydrogels, i.e., dense and lacking a large pore size facilitated in tissue and blood vessels. Therefore, several traditional methods have been attempted to increase the pore size of hydrogels in the early stage. One is the salting method, but we found that we cannot get the ideal connecting hole. The other method is that the hydrogel is prepared and then freeze-dried. Although the pore size increased with the decrease in polymer content, the hydrogel cannot form after a certain degree of reduction. Therefore, we freeze-dried the polymer solution before gelating to obtain sponge-like scaffolds and photo crosslinking for the macroporous architecture. Importantly, low polymer concentration will not affect crosslinking. As shown in Figure 2E, the pore size of L-PGS-P (200–500 μm) was larger than that of PGS-P (2–5 μm). According to the previous consensus on pore classification, pores > 100 μm in tissue regeneration substitutes are defined as macropores. Such a macroporous structure is good for cell attachment, migration, and bone ingrowth into scaffolds (Jones and Hench, 2004).

After successfully preparing functional hydrogels, we needed to inspect the functional properties of the material's cellular level *in vitro* and *in vivo* animal bone defect models. This was done to explore the conceivable clinical applications of bio-artificial multiporous matrices. First, scaffolds were evaluated for the effect of the fabrication method on cellular infiltration *in vitro*. After the endothelial cells were seeded on the surface of the materials, the number of cells entering the interior of the scaffolds was observed using fluorescence staining. Many cells entered the L-PGS-P compared with that in PGS-P, which proved that the macroporous structure produced was sufficient for the cells to enter from the surface to the interior.

Generally, the induction and promotion of the osteogenic ability of stem cells are crucial steps for continuous mineralization (Ramaswamy et al., 2008). In this study, the adhesion, proliferation, ALP, activity, and osteogenic gene expression of stem cells were investigated. First, we inspected the adhesion of BMSCs to PGS-P and L-PGS-P scaffolds (Figure 3A), which was related to the introduction of RGD. At the same time, the osteogenic gene was significantly up-regulated in the material groups at 3 and 7 days, and the osteogenic properties of the L-PGS-P group were better than those of the PGS-P group (Figure 4).

After the scaffolds' implantation, there will be an inflammatory phase, vascular phase, and subsequent bone tissue repair stage. In the process of vascular regeneration, growth starts from the blood vessels around the scaffold. The abundant blood vessels and high perfusion of blood imply that more bone cells will be delivered and nutrient delivery for the new bone will improve, which will accelerate bone regeneration (Liu et al., 2017). *In vitro* tests showed that the tube forming ability of the material group was significantly improved compared with the control group (Figure 3D), which was owing to the release of VEGF after grafting, and the tube forming ability of L-PGS-P was more obvious than that of PGS at the same time owing to the accelerated degradation. *In vivo* vascularization experiments confirmed that the PGS-based biological scaffold promotes vascularization compared with the control group. At the same time, as designed in this study, the L-PGS-P scaffold had a faster bone tissue repair rate and more vascular tissue growth. This study verified that a functional biomaterial based on PGS has excellent properties of promoting osteogenesis and angiogenesis.

However, there are some limitations to this study. One is that the *in vivo* experiments are based on a 2.5-mm size defect, which may not be critical enough to fully inspect the scaffold's abilities since Sprague–Dawley rats may be able to achieve repair without any assistance. Another limitation is that we did not evaluate the effect of different pore sizes on vascularization and bone regeneration. Therefore, it is unclear if the positive effect was achieved by the ingredients of the compound or the specific pore size and structure. In a future study, the PGS-P scaffold should be examined with a more critical bone defect, and scaffolds with different pore sizes should be compared.

## Conclusions

In previous studies, PGS that supported greater rBMSC adhesion, signaling, and proliferation was developed, proving its application in tissue engineering. However, the hydrophobicity of PGS makes it difficult to prepare hydrogels. Moreover, even if many methods have been applied to improve hydrophilicity, the pore size of the hydrogel obtained owing to the presence of hydrophobic segments is not conducive to subsequent tissue replacement after cell invasion. In this study, bone-mimetic porous scaffolds consisting of PGS biomaterials and proangiogenic VEGF mimetic peptide caused a significant increase in vascularization and bone regeneration by effectively preparing the interconnected macroporous structure hydrogel and endowing vascular function. Lyophilization before crosslinking through light triggering techniques proved that it could produce macropore structures similar to spinning and has the capability to increase cell infiltration and vascularization. *In vitro* and *in vivo* tests, compared with the control group, showed the material group exhibited better ability of vascularization and osteogenesis. More importantly, L-PGS-P recruited more HUVECs and significantly increased the formation of blood vessels because of promoting cell invasion. Compared with the PGS-P group, the L-PGS-P group produced bone faster, which appeared simultaneously in the center and around the defect. These collective findings support the use of a PGS-based bio-artificial multiporous matrix as potential bone repair materials.

## Data Availability Statement

The original contributions generated for the study are included in the article/Supplementary Material, further inquiries can be directed to the corresponding author/s.

## Ethics Statement

The animal study was reviewed and approved by the Animal Research Committee of the Sixth People's Hospital, Shanghai Jiao Tong University.

## Author Contributions

BL contributed to conceptualization, formal analysis and investigation, methodology and project administration, and writing of the original draft. QS contributed to data curation, methodology, and project administration. JX curated the data. J-GX and Y-MC contributed to supervision, validation, reviewing, and editing the paper. All authors contributed to the article and approved the submitted version.

## Conflict of Interest

The authors declare that the research was conducted in the absence of any commercial or financial relationships that could be construed as a potential conflict of interest.

## Abbreviations

HUVEC: human umbilical vein endothelial cell
PBS: phosphate-buffered saline
PEG: polyethylene glycol
PGS: poly (glycerol sebacate)
rBMSCs: rat bone marrow stromal cells
SEM: scanning electron microscopy
VEGF: vascular endothelial growth factor.
